# Relationship of Thyroid Volume and Function with Ankle-Brachial Index, Toe-Brachial Index, and Toe Pressure in Euthyroid People Aged 18–65

**DOI:** 10.3390/medicina60091445

**Published:** 2024-09-04

**Authors:** Grzegorz K. Jakubiak, Natalia Pawlas, Małgorzata Morawiecka-Pietrzak, Jolanta Zalejska-Fiolka, Agata Stanek, Grzegorz Cieślar

**Affiliations:** 1 Department of Pharmacology, Faculty of Medical Sciences in Zabrze, Medical University of Silesia, 41-800 Zabrze, Poland; n-pawlas@wp.pl; 2 Department of Pediatrics, Faculty of Medical Sciences in Zabrze, Medical University of Silesia, 41-800 Zabrze, Poland; morawieckam@gmail.com; 3 Department of Biochemistry, Faculty of Medical Sciences in Zabrze, Medical University of Silesia, 41-800 Zabrze, Poland; jzalejskafiolka@sum.edu.pl; 4 Department of Internal Medicine, Angiology and Physical Medicine, Faculty of Medical Sciences in Zabrze, Medical University of Silesia, 41-902 Bytom, Poland; astanek@tlen.pl (A.S.); cieslar1@tlen.pl (G.C.)

**Keywords:** thyroid volume, thyrotropin, triiodothyronine, thyroxine, ankle-brachial index, toe-brachial index, toe pressure, subclinical cardiovascular dysfunction

## Abstract

*Background and Objectives*: The interrelationship between thyroid function and the state of the cardiovascular system has been investigated both in preclinical and human studies. However, it remains unclear whether there is any association between thyroid hormones and features of subclinical cardiovascular dysfunction in euthyroid patients. *Material and Methods*: This study involved 45 people (females: 57.8%) with no thyroid disease who, during planned hospitalization, underwent thyroid ultrasound, determination of biochemical parameters of thyroid function, and measurement of ankle-brachial index (ABI) and toe-brachial index (TBI). People with signs of acute illness or a deterioration of their health were excluded. *Results:* Significant correlations were found between free triiodothyronine (FT3) and several parameters of both ABI (R = 0.347; *p* = 0.019 for the mean ABI taken from right side and left side values) and TBI (R = 0.396; *p* = 0.007 for the mean TBI taken from right side and left side values), as well as the maximal toe pressure (TP) taken from right side and left side values (R = 0.304; *p* = 0.045). Thyrotropin (TSH) was shown to be significantly correlated only with the maximal TBI value (taken from right side and left side values) (R = 0.318; *p* = 0.033), whereas free thyroxin (FT4) was shown to be significantly correlated only with the minimal TBI value (taken from right side and left side values) (R = 0.381; *p* = 0.01). Thyroid volume (TV) was shown to be correlated with TP (R = 0.4; *p* = 0.008 for the mean TP taken from right side and left side values) and some parameters of TBI value (R = 0.332; *p* = 0.028 for the mean TBI taken from right side and left side values), but no significant correlations were found between TVand ABI parameters. Patients with a mean ABI value ≤ 1.0 or a mean TBI value ≤ 0.75 have lower TSH, FT3, FT4, and TV than the rest of the study population, but the difference was statistically significant only for FT3. *Conclusions:* Even in a population of euthyroid patients with no diagnosed thyroid disease, there are some significant correlations between the volume and function of the thyroid gland and the selected features of subclinical cardiovascular dysfunction such as ABI and TBI.

## 1. Introduction

Cardiovascular diseases (CVDs) are one of the most crucial problems for public health worldwide [1] because they create the leading cause of morbidity and mortality in many countries, as well as because of the increasing occurrence in the world population of different cardiovascular risk factors such as obesity [2,3], metabolic syndrome [4,5], dyslipidemia [6], diabetes mellitus [7], and arterial hypertension [8].

Although both pharmacological and non-pharmacological possibilities and strategies of treatment of clinically overt CVD developed significantly in recent decades, the development and search for methods of identification of cardiovascular dysfunction at the subclinical stage seems to be a crucial problem currently because it may help to select patients who should be under more intensive control and prevention, as well as to introduce the appropriate treatment methods at the optimal moment [9]. Among the methods considered to be useful in the assessment of subclinical cardiovascular dysfunction, such as ankle-brachial index (ABI) [10], endothelial function assessment by flow-mediated dilation (FMD) [11], arterial stiffness assessment by pulse-wave velocity [12], intima media thickness (IMT) measurement [13], and coronary artery calcium score [14,15] should be mentioned.

ABI is defined as systolic blood pressure at the level of the ankle divided by systolic blood pressure at the level of the arm [16]. ABI is the basic tool for the diagnosis of peripheral arterial disease (PAD) in the form of chronic lower extremities ischemia [17]. Moreover, ABI signifies atherosclerosis in other vascular areas and can act as a predictive marker for cardiovascular events, even without PAD symptoms [18]. Toe-brachial index (TBI) is defined analogously as systolic blood pressure at the level of the toe (toe pressure, TP) divided by systolic blood pressure at the level of the arm [16]. TBI is a valuable addition to ABI measurement, especially in situations when ABI can be falsely increased due to calcification of the medium layer of the arterial wall (patients with diabetes, chronic end-stage renal disease, or patients with long-lasting immunosuppressive treatment) [17].

More and more attention has been paid to the issue of the interrelationship between thyroid function and the state of the cardiovascular system [19]. Thyroid hormones may affect the cardiovascular system according to different mechanisms. Thyroid hormones influence cardiomyocytes directly through regulation of myosin heavy chains [20], modification of sarcoplasmic reticulum calcium ATPase (SERCA) activity [21], as well as ion channels [22] and intracellular signal transduction pathways [23]. Thyroid hormones have direct effects on endothelial cells [24] and vascular smooth muscle cells [25]. Thyroid hormones modify some cardiovascular risk factors, such as blood pressure or lipid profile [26]. Disturbances in thyroid function also influence coagulation and fibrinolysis [27,28].

In human studies, the relationship between thyroid function and cardiovascular morbidity and mortality has also been analyzed [29,30]. The relationship between abnormal thyroid function at the time of myocardial infarction and subsequent adverse cardiovascular outcomes has been discussed [31]. Interestingly, according to the meta-analysis performed by Aziz et al., thyroxin therapy in subjects with subclinical hypothyroidism significantly decreases carotid intima-media thickness [32]. However, it remains unclear whether there is any association between thyroid hormones and volume and features of subclinical cardiovascular dysfunction in euthyroid patients with no overt CVD.

In a previously published article, we showed that there are certain relationships between thyroid volume and function with anthropometric measurements and body composition analysis, as well as the diagnosis of metabolic syndrome in euthyroid patients aged 18–65 [33]. Currently, we analyzed the same group of patients using data related to the selected parameters of cardiovascular system assessment such as ABI, TBI, and TP.

The purpose of this study was to investigate the relationship between the parameters of thyroid assessment and selected parameters related to the function of the cardiovascular system (ABI, TBI, and TP) in euthyroid patients aged 18–65.

## 2. Materials and Methods

### 2.1. Study Population

Data from the medical records of patients hospitalized in the Department of Internal Medicine, Angiology and Physical Medicine of the Medical University of Silesia in Katowice (Poland) from June 2022 to October 2023 was retrospectively analyzed. Only patients with no features of acute illness or exacerbation of chronic diseases participated in the study. People with diagnosed thyroid disease were excluded even if, according to the currently performed test, they were classified as euthyroid. The inclusion and exclusion criteria are described thoroughly in our previous paper, in which the same patients participated [33].

### 2.2. Laboratory Tests

Basic laboratory tests were performed in blood samples collected in the morning between 8 and 10 a.m., at least fourteen hours after the last meal. The following parameters were determined to assess thyroid function: thyrotropin (TSH), free triiodothyronine (FT3), and free thyroxine (FT4). Autoantibodies tests used in the diagnosis of thyroid diseases have not been performed, and thyroglobulin serum concentration has not been measured.

FT3, FT4, and TSH serum concentration measurements were performed in the Laboratory of Specialist Hospital No. 2 in Bytom by the electrochemiluminescence assay (ECLIA) using the Elecsys^®^ reagent kits (Roche Diagnostics GmbH, Mannheim, Germany) using calibration principles according to the manufacturer’s instructions.

### 2.3. Thyroid Ultrasound

A thyroid ultrasound examination was performed using a Samsung RS80 EVO device (Samsung Medison Co., Ltd., Seoul, Korea) with a linear probe (LA4-18B) by the same physician in each case. The location of the thyroid gland and the echogenicity of the thyroid parenchyma were assessed, as were the sizes of both lobes (in three dimensions) and the thickness of the isthmus in each examination. The estimated volume of the gland was calculated by taking into account three measurements obtained for each lobe. The thyroid gland was also assessed for focal lesions, which were described in each case in terms of their size and structure.

### 2.4. Additional Derivatives of Thyroid Parameters

For better understanding the nature of the relationship between thyroid function and the assessed vascular parameters, we performed additional correlation analysis for selected parameters calculated based on FT3, FT4, TSH, and TV, such as:

(1) FT3/FT4 as a marker of peripheral conversion of triiodothyronine to thyroxine [34,35,36];
(2) FT4 × TSH as a marker of peripheral resistance for thyroid hormone activity as well as a marker of thyrotroph sensitivity to thyroid hormone [37,38,39];
(3) FT4 × TSH × TV analogously as FT4 × TSH but taking into consideration also TV value;
(4) FT3 × FT4 × TSH × TV analogously as previously mentioned parameters but taking into consideration both free thyroid hormones.

According to the best of our knowledge, properties of such parameters as FT4 × TSH × TV and FT3 × FT4 × TSH × TV have not been described in the literature so far. We decided to use such products of simple thyroid parameters, taking into account literature reports related to FT4 × TSH as a marker of peripheral resistance to thyroid hormones as mentioned above, the physiological function of the hypophysis-thyroid axis, as well as the mathematical properties of the product of several variables. It is known that endogenic thyrotropin influences positive thyroid gland volume [40]. Thyroid hormones influence thyrotrophs through negative feedback. Resistance of peripheral tissues and thyrotrophs for thyroid hormones therefore can stimulate TSH excretion. In view of the above reasoning, it can be expected that with the increasing resistance of peripheral tissues to thyroid hormones (measured by FT4 × TSH described in the literature), there will be a tendency for the volume of the thyroid gland (TV) to increase, which will also translate into an increase in the value of the product of these two parameters (FT4 × TSH × TV). Similar reasoning, but taking into account both thyroid hormones, leads to the use of the expression FT3 × FT4 × TSH × TV.

### 2.5. Ankle-Brachial Index (ABI)

ABI was measured by Dopplex^®^ Ability Automatic ABI System (Huntleigh Healthcare Ltd., Cardiff, UK). Each examination was performed in thermal comfort, in a supine position, after at least 10 minutes of rest in a supine position immediately before the measurement. ABI was calculated by division of the ankle pressure by the highest value of arm pressure for the right side and the left side, respectively.

Analyzing collected data, we presented the ABI value for each patient as ABI measured on the right side (ABI right) and ABI measured on the left side (ABI left), as well as the maximal ABI value (ABI max), the minimal ABI value (ABI min), and the mean ABI value (ABI mean), taking into consideration both ABI right and ABI left.

### 2.6. Toe Pressure (TP) and Toe-Brachial Index (TBI)

TP was measured by Dopplex^®^ DMX Digital Doppler (Huntleigh Healthcare Ltd., Cardiff, UK) directly after the measurement of ABI. TBI was calculated by division of TP by the higher value of the arm pressure, for the right side and the left side appropriately.

Similarly to ABI, we presented the TBI value for each patient as TBI measured on the right side (TBI right) and TBI measured on the left side (TBI left), as well as the maximal TBI value (TBI max), the minimal TBI value (TBI min), and the mean TBI value (TBI mean), taking into consideration both TBI right and TBI left. TP has been presented according to the analogous conception.

### 2.7. Statistical Analysis

The Shapiro-Wilk test, analysis of the distribution parameters, and visual assessment of the histogram were used to check the compliance of the distribution of the quantitative variables with the normal distribution.

The values of normally distributed quantitative variables were presented as mean and standard deviation (SD). The values of not normally distributed quantitative variables were presented as median and the first and third quartiles values (Q1; Q3). Ranges for all quantitative variables were also presented. The values of the qualitative variables were presented in the form of the number (and the percentage) of a given variant.

Spearman’s rank correlation test was used to examine the correlation between the measured vascular parameters and the thyroid volume and the thyroid function parameters. To compare the significance of differences between ABI, TBI, and TP bilaterally, the Student’s *t*-test for dependent variables was used for variables whose distribution did not differ significantly from the normal distribution (after previous checking that the distribution of differences does not differ significantly from the normal distribution), and the Wilcoxon test was used for variables whose distribution differed significantly from the normal distribution. To compare the significance of differences between ABI, TBI, and TP between given subgroups, a Student’s *t*-test for independent variables was used for variables whose distribution did not differ significantly from the normal distribution, and the U Mann-Whitney test was used for variables whose distribution differed significantly from the normal distribution. *p* < 0.05 was considered statistically significant.

Statistical analysis was performed using TIBCO Software Inc. (Palo Alto, CA, USA, 2017) Statistica (data analysis software system), version 13.

### 2.8. Ethical Aspects

An inquiry was submitted to the Bioethics Committee of the Medical University of Silesia in Katowice. The response was that a study involving a retrospective analysis of medical records does not require approval of the Bioethics Committee (6 February 2024, decision no. BNW/NWN/0052/KB/19/24).

## 3. Results

### 3.1. Study Population: General Characteristics and Thyroid Parameters

45 patients with a median age of 49.9 years (37.6; 56.2) were included in the final analysis (females: 57.8%). The complete description of the study population, as well as the results of thyroid ultrasound and the biochemical parameters of thyroid function, are presented in the previous publication [33]. No one in the study population was diagnosed with atrial fibrillation, chronic kidney disease, anemia, active neoplastic disease, or autoimmunity.

### 3.2. Ankle Brachial Index

In two patients (4.4%), the minimal ABI value was not higher than 0.9, whereas in four patients (8.9%), the minimal ABI value was higher than 0.9 and not higher than 1.0. It is worth noting that in no patient, ABI was higher than 1.3. No significant differences have been found between median ABI right and median ABI left (*p* = 0.326).

Table 1 shows descriptive statistics for ABI values in the study population. All parameters were not normally distributed.

### 3.3. Toe Pressure and Toe-Brachial Index

In 15 patients (33.3%), the minimal TBI value was not higher than 0.7, whereas in 7 patients (15.6%), both TBI right and TBI left were lower than 0.7. No significant differences have been found between mean TBI right and mean TBI left (*p* = 0.649), as well as between mean TP right and mean TP left (*p* = 0.57). There is missing data for TP in one patient (only TBI values have been written in the result in the medical history).

Table 2 shows descriptive statistics for TP and TBI values in the study population. All parameters were normally distributed.

As expected, the mean TBI value was strongly correlated with the mean TP value (R = 0.838; *p* < 0.001). On the other hand, no significant correlation was found between the mean TBI value and the mean ABI value (R = 0.285, *p* = 0.058), which confirms that TBI measurement is a valuable complementary method for ABI measurement.

### 3.4. Correlation between the Thyroid Function Parameters and Vascular Parameters

In terms of biochemical parameters of thyroid function, FT3 was found to be more closely correlated with the assessed vascular parameters than TSH and FT4. FT3 was significantly correlated with ABI parameters with exception for minimal ABI values (taken from right and left side values), as well as with TBI parameters with exception for the left side TBI value. TSH was significantly correlated only with the maximal TBI value (taken from right and left side values), whereas FT4 was significantly correlated only with the minimal TBI value.

Table 3 shows a full description of Spearman’s rank correlation coefficient for the relationship between biochemical parameters of thyroid function and ABI, TBI, and TP values.

### 3.5. Correlation between the Thyroid Volume and Vascular Parameters

In the study population, thyroid volume was significantly correlated with all parameters related to TP, as well as mean, maximal, and minimal TBI values, but not with right side and left side TBI values considered separately. Table 4 shows the full description of Spearman’s rank correlation coefficient for the relationship between thyroid volume and ABI, TBI, and TP values.

### 3.6. Differences between Subgroups According to the Vascular Parameters

We analyzed differences in thyroid volume and biochemical parameters between subpopulation with lower values of ABI or TBI and the rest of the study participants. According to our results, patients with a mean ABI not exceeding 1.0 or a mean TBI not exceeding 0.75 have lower mean TSH, FT3, and FT4 serum concentrations, as well as lower TV, although only for FT3 the difference was statistically significant. Table 5 shows a full description of differences between subgroups according to the mentioned above criterion.

### 3.7. Correlation between Additional Derivatives of Thyroid Parameters and Vascular Parameters

The results of the Spearman’s rank correlation test for the mentioned above derivatives from thyroid parameters and the measured vascular parameters are presented in Table 6.

According to data from Table 6, no significant correlation has been found between FT3/FT4 and ABI parameters, FT4 × TSH and ABI parameters, FT4 × TSH × TV and ABI parameters, as well as FT3 × FT4 × TSH × TV and ABI parameters. The strongest correlations have been found between FT4 × TSH × TV and TBI max, as well as FT3 × FT4 × TSH × TV and TBI max, and similarly for TBI mean.

We would like to emphasize that we do not claim that such parameters as FT4 × TSH × TV and FT3 × FT4 × TSH × TV used per se have any significance for clinical practice because this would require separate studies. We only believe that the results we obtained by examining the correlation of these parameters with the parameters used to assess the cardiovascular system are interesting from the point of view of the pathophysiology of the relationship between the thyroid gland and the cardiovascular system, which is the subject of this paper.

**Figure 1:**
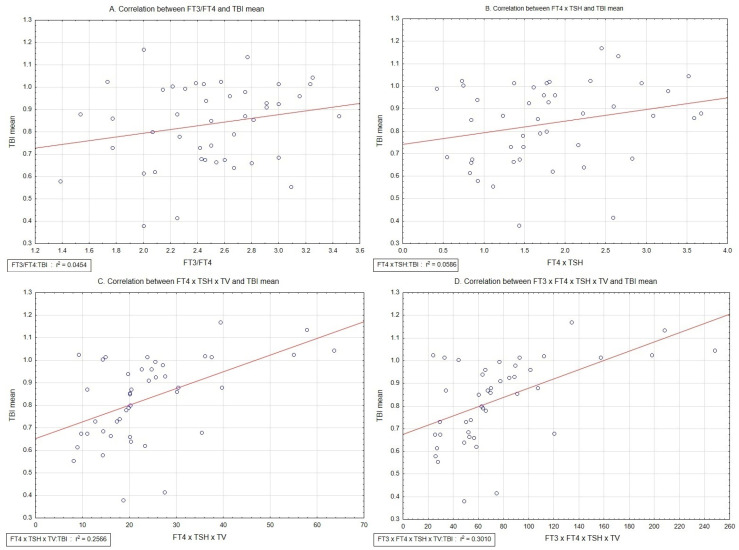
Correlations between FT3/FT4 and TBI mean (**A**), FT4 × TSH and TBI mean (**B**), FT4 × TSH × TV and TBI mean (**C**), and FT3 × FT4 × TSH × TV and TBI mean (**D**). Abbreviations: TSH—thyrotropin; FT3—free triiodothyronine; FT4—free thyroxine; TV—thyroid volume; TBI mean—mean toe-brachial index value (taken from right side and left side values).

Correlations between TBI mean and defined above derivatives from thyroid parameters are graphically presented in Figure 1.

### 3.8. Additional Subgroups Analysis According to Comorbidities

For better understanding the results we had obtained, additional analysis in subgroups according to such factors as arterial hypertension, diabetes mellitus, obesity, and metabolic syndrome was performed.

#### 3.8.1. Arterial Hypertension

No significant differences were found between patients with and without arterial hypertension in terms of ABI, TBI, and TP values as well as in terms of biochemical parameters of thyroid function (FT3, FT4, TSH). Interestingly, TV was found to be significantly higher in patients with arterial hypertension in comparison to patients without arterial hypertension. Table 7 shows a full description of differences between subgroups according to the diagnosis of arterial hypertension.

#### 3.8.2. Diabetes Mellitus

No significant differences have been found between patients with diabetes or prediabetes (9 patients) and patients without diagnosed abnormalities in carbohydrates metabolism (36 patients) in terms of ABI, TBI, and TP, as well as in terms of all thyroid parameters. Table 8 shows a full description of differences between subgroups according to the diagnosis of diabetes or prediabetes.

#### 3.8.3. Overweight and Obesity

No significant differences were found between patients with normal weight or underweight (BMI < 25.0 kg/m^2^) and patients with overweight or obesity (BMI ≥ 25.0 kg/m^2^) in terms of ABI and TBI. All TP parameters were found to be significantly higher in overweight or obese patients than in normal-weight or underweight patients. Table 9 shows a full description of differences between subgroups according to the BMI value. No significant difference has been found in terms of FT3 and FT4, whereas TSH serum concentration was significantly higher in normal-weight or underweight patients and TV was significantly lower in normal-weight or underweight patients, as described in detail in our previous publication, so it was not repeated in Table 9 [33].

#### 3.8.4. Metabolic Syndrome

No significant differences have been found between patients with metabolic syndrome and patients without metabolic syndrome in terms of ABI, TBI, and TP. Table 10 shows a full description of differences between subgroups according to the diagnosis of metabolic syndrome. TV was found to be significantly higher in patients with metabolic syndrome, whereas no significant difference was found in terms of biochemical parameters of thyroid function, as was described in detail in our previous publication, so it was not repeated in Table 10 [33].

## 4. Discussion

According to the results we obtained, thyroid assessment parameters show some correlations with parameters of subclinical cardiovascular dysfunction (ABI, TBI, TP) in patients without diagnosed thyroid disease. It should be emphasized that the group we examined consisted of people in good health without diagnosed cancer, autoimmune diseases, or chronic inflammatory diseases. It is also worth noting that in the study group, the diagnosis of diabetes, hypertension, obesity, or metabolic syndrome was not associated with a significant effect on the ABI and TBI values, which suggest an early and relatively uncomplicated stage of the mentioned diseases. FT3 serum concentration correlates significantly with ABI (right side value, left side value, mean value, and maximal value, but not with minimal value), with maximal TP value (but not other values for these parameters), as well as with TBI (maximal value, minimal value, mean, and right side value, but not with left side value). Moreover, TSH serum concentration correlates significantly only with maximal TBI values (but not with any other assessed vascular parameter), and FT4 correlates significantly with minimal TBI values (but not with any other assessed vascular parameters). TV was shown to be significantly correlated with all TP parameters, as well as with TBI (minimal, maximal, and mean), but not with ABI. In the study population, patients with lower values of ABI and TBI (defined as mean ABI not exceeding 1.0 or mean TBI not exceeding 0.75) have lower values of TV, as well as TSH, FT3, and FT4 serum concentration, but only for FT3 the difference was confirmed to be statistically significant. Moreover, multiplicative combinations of FT3, FT4, TSH, and TV were shown to be strongly associated with all TBI parameters and TP parameters (with exception for minimal TP), although for TP the correlation was weaker as well as not to be correlated with ABI parameters.

The issue of the relationship between thyroid hormones and ABI has already been undertaken by some researchers. In a study performed on the Chinese population, it was found that patients in the highest quartile of FT3 serum concentration have a decreased risk for PAD diagnosis (defined as ABI < 0.9), which is in accordance with our results showing a significant positive correlation between FT3 and ABI. On the other hand, in this study performed by Wang et al., a significant correlation between ABI and FT3/FT4 has also been found, which has not been confirmed in results based on the data collected in our study [41]. According to Zhuang et al. a positive predictive value for thyroxine serum concentration in PAD diagnosis (defined as ABI < 1.0 or ABI > 1.4) (OR 1.45, 95% CI 1.21–1.75 or OR 1.54, 95% CI 1.14–2.08 depending on statistical analysis methodology) [42]. In a large study performed in Germany (5818 individuals), no significant association was found between TSH serum concentration and ABI value, similarly to our study [43].

Interesting results have been found in a study conducted by Ozkan and colleagues from Turkey. Patients with differentiated thyroid cancer both before and after thyroid hormone withdrawal (a short-term overt hypothyroid phase) had decreased ABI compared to the control group. In a study group before the hypothyroid phase, no significant correlations have been found between ABI and hormones (TSH, FT3, FT4), whereas after treatment associated with hypothyroid phase ABI was significantly correlated with TSH, FT3, and FT4, suggesting a cause-and-effect relationship between changes in thyroid function and ABI value [44]. Li et al. presented results of an observational study in which effects of the treatment of hyperthyroidism in the course of Graves’ disease have been assessed. At baseline, a significant negative correlation was found between ABI value and FT4 (r = −0.335, *p* < 0.001). After the anti-thyroid treatment, FT4 serum concentration significantly decreased as expected. Interestingly, the decrease in FT4 serum level was significantly correlated with the increase in ABI value (r = − 0.406, *p* = 0.021) [45]. The results of the two mentioned studies are difficult to compare with our study because our study population consists only of euthyroid patients with no diagnosed thyroid disease. Moreover, the changes in ABI associated with modification of thyroid gland activity in relatively short time suggest that the relationship between thyroid function and lower extremities blood flow is at least partially not only the effect of permanent vascular remodeling but also of transient pharmacological activity of thyroid hormones. It should be emphasized that the true understanding of the mechanism of influence of thyroid function on vascular function requires further research.

Although no study has been found in which the association between TV and ABI would be analyzed, the issue of the relationship between thyroid morphology and some aspects of the function of the cardiovascular system has already been analyzed by other researchers. In the study performed by Aydoğan et al. it was shown that euthyroid patients with thyroid focal lesions have significantly higher thyroid volume (11.87 mL vs. 9.43 mL; *p* = 0.005) and simultaneously significantly higher values of pulse wave velocity (6.5 m/s vs. 5.30 m/s; *p* < 0.001), which is also one of the parameters used to assess subclinical cardiovascular dysfunction [46].

Preparing this manuscript, we found no study in the commonly available literature in which a relationship of thyroid morphology and function with TBI and TP would be analyzed, so in this aspect our study may be considered to be innovative as well as an interesting direction for further research. Because of the small number of study participants, our results should be considered as a preliminary analysis and suggestion for further investigation. On the other hand, it should be emphasized that despite the small study group, our results on the relationship between thyroid function and ABI give similar conclusions as certain studies performed by other research groups.

### Strengths and Limitations of the Study

The presented study has some limitations. First of all, conclusions can be drawn only about the existence of certain relationships at a specific point in time because of the cross-sectional nature of the study. However, it is not possible to conclude about possible cause-and-effect relationships. Moreover, it would be worth repeating the study on a larger group of participants because the relatively small study group is another disadvantage of the presented research. It should also be emphasized that autoantibodies used in the diagnosis of thyroid disease and thyroglobulin serum levels have not been determined in the study population.

This study also has some strengths. The most significant strength of our study is the simultaneous assessment of TV, TSH, FT3, and FT4, as well as ABI and TBI. TBI is usually not measured in patients with normal ABI, so measurement of both parameters simultaneously is an important advantage, mainly taking into consideration the retrospective nature of our study. Similarly to our previous publication, which was based on the same study population, it would be worth emphasizing that all data were collected from the patients during their hospitalization. This means that the information about the patients was not based only on interview data but came from currently performed diagnostic procedures. There were also a few missing data points in the collected material (value of thyroid volume in one patient and value of toe pressure in one patient), which elucidates that although it is a retrospective study, the data has been collected thoroughly in analyzed medical histories.

## 5. Conclusions

In the presented study, some significant relationships have been found between biochemical parameters of thyroid function and thyroid volume and parameters of subclinical cardiovascular dysfunction such as ABI, TBI, and TP in euthyroid patients with no diagnosed thyroid disease.

According to the best of our knowledge, the relationship of thyroid volume and function with TP and TBI has not been studied so far in a similar study population. However, we present the results of the retrospective cross-sectional study on a small population, so our finding should be considered to be only a preliminary result and an inspiration as an interesting direction for further research and verification in prospective observation on a larger cohort.

## Figures and Tables

**Table 1:** Descriptive statistic of ankle-brachial index (ABI) values in the study population.

Parameter	N	Median (Q1; Q3)	Range
ABI right	45	1.15 (1.08; 1.21)	0.51–1.3
ABI left	45	1.15 (1.1; 1.2)	0.8–1.3
ABI mean	45	1.16 (1.1; 1.22)	0.66–1.29
ABI max	45	1.17 (1.12; 1.23)	0.8–1.3
ABI min	45	1.13 (1.08; 1.2)	0.51–1.28

Abbreviations: ABI right—ankle-brachial index measured on the right side; ABI left—ankle-brachial index measured on the left side; ABI mean—mean ankle-brachial index value (taken from right side and left side values); ABI max—maximal ankle-brachial index value (taken from right side and left side values); ABI min—minimal ankle-brachial index value (taken from right side and left side values).

**Table 2:** Descriptive statistics of toe pressure (TP) and toe-brachial index (TBI) values in the study population.

Parameter	N	Mean (SD)	Range
TP right [mmHg]	44	102.0 (25.81)	52.0–184.0
TP left [mmHg]	44	103.75 (24.7)	48.0–155.0
TP mean [mmHg]	44	102.88 (23.14)	50.0–167.0
TP max [mmHg]	44	110.61 (26.7)	52.0–184.0
TP min [mmHg]	44	95.14 (21.04)	48.0–150.0
TBI right	45	0.83 (0.2)	0.3–1.29
TBI left	45	0.84 (0.2)	0.4–1.18
TBI mean	45	0.83 (0.18)	0.38–1.17
TBI max	45	0.9 (0.21)	0.43–1.29
TBI min	45	0.77 (0.17)	0.3–1.05

Abbreviations: TP right—toe pressure measured on the right side; TP left—toe pressure measured on the left side; TP mean—mean toe pressure value (taken from right side and left side values); TP max—maximal toe pressure value (taken from right side and left side values); TP min—minimal toe pressure value (taken from right side and left side values); TBI right—toe-brachial index measured on the right side; TBI left—toe-brachial index measured on the left side; TBI mean—mean toe-brachial index value (taken from right side and left side values); TBI max—maximal toe-brachial index value (taken from right side and left side values); TBI min—minimal toe-brachial index value (taken from right side and left side values).

**Table 3:** The results of the Spearman’s rank correlation test between the biochemical parameters of thyroid function assessment and vascular parameters.

Parameter	N	TSH		FT3		FT4	
		*R*	*p*	*R*	*p*	*R*	*p*
ABI right	45	−0.266	0.077	0.334	0.025	0.2	0.189
ABI left	45	−0.071	0.641	0.314	0.036	0.269	0.074
ABI mean	45	−0.172	0.258	0.347	0.019	0.237	0.117
ABI max	45	−0.118	0.44	0.409	0.005	0.225	0.137
ABI min	45	−0.23	0.129	0.273	0.07	0.244	0.106
TP right	44	−0.078	0.616	0.261	0.088	0.212	0.168
TP left	44	0.117	0.448	0.153	0.32	0.101	0.513
TP mean	44	0.022	0.886	0.253	0.098	0.198	0.199
TP max	44	0.133	0.391	0.304	0.045	0.068	0.66
TP min	44	−0.112	0.468	0.137	0.373	0.293	0.053
TBI right	45	0.16	0.294	0.411	0.005	0.225	0.138
TBI left	45	0.289	0.054	0.265	0.079	0.167	0.273
TBI mean	45	0.239	0.114	0.396	0.007	0.244	0.106
TBI max	45	0.318	0.033	0.394	0.007	0.11	0.472
TBI min	45	0.139	0.362	0.312	0.037	0.381	0.01

Abbreviations: ABI right—ankle-brachial index measured on the right side; ABI left—ankle-brachial index measured on the left side; ABI mean—mean ankle-brachial index value (taken from right side and left side values); ABI max—maximal ankle-brachial index value (taken from right side and left side values); ABI min—minimal ankle-brachial index value (taken from right side and left side values); TP right—toe pressure measured on the right side; TP left—toe pressure measured on the left side; TP mean—mean toe pressure value (taken from right side and left side values); TP max—maximal toe pressure value (taken from right side and left side values); TP min—minimal toe pressure value (taken from right side and left side values); TBI right—toe-brachial index measured on the right side; TBI left—toe-brachial index measured on the left side; TBI mean—mean toe-brachial index value (taken from right side and left side values); TBI max—maximal toe-brachial index value (taken from right side and left side values); TBI min—minimal toe-brachial index value (taken from right side and left side values). Statistically significant correlations are marked in red.

**Table 4:** The results of the Spearman’s rank correlation test between thyroid volume and vascular parameters.

Parameter	N	Thyroid Volume	
		*R*	*p*
ABI right	44	0.2	0.192
ABI left	44	0.166	0.282
ABI mean	44	0.191	0.215
ABI max	44	0.167	0.278
ABI min	44	0.192	0.212
TP right	43	0.352	0.021
TP left	43	0.32	0.037
TP mean	43	0.4	0.008
TP max	43	0.386	0.01
TP min	43	0.342	0.025
TBI right	44	0.293	0.053
TBI left	44	0.236	0.124
TBI mean	44	0.332	0.028
TBI max	44	0.301	0.047
TBI min	44	0.311	0.04

Abbreviations: ABI right—ankle-brachial index measured on the right side; ABI left—ankle-brachial index measured on the left side; ABI mean—mean ankle-brachial index value (taken from right side and left side values); ABI max—maximal ankle-brachial index value (taken from right side and left side values); ABI min—minimal ankle-brachial index value (taken from right side and left side values); TP right—toe pressure measured on the right side; TP left—toe pressure measured on the left side; TP mean—mean toe pressure value (taken from right side and left side values); TP max—maximal toe pressure value (taken from right side and left side values); TP min—minimal toe pressure value (taken from right side and left side values); TBI right—toe-brachial index measured on the right side; TBI left—toe-brachial index measured on the left side; TBI mean—mean toe-brachial index value (taken from right side and left side values); TBI max—maximal toe-brachial index value (taken from right side and left side values); TBI min—minimal toe-brachial index value (taken from right side and left side values). Statistically significant correlations are marked in red.

**Table 5:** Differences between patients with mean ankle-brachial index (ABI) values not exceeding 1.0 or mean toe-brachial index (TBI) values not exceeding 0.75 and the rest of the study population in terms of thyroid volume and function.

Parameter	ABI Mean ≤ 1.0 or TBI Mean ≤ 0.75		ABI Mean > 1.0 and TBI Mean > 0.75		*p*
	N	Mean (SD)/Median (Q1; Q3)	N	Mean (SD)/Median (Q1; Q3)	
TSH [µIU/mL]	18	1.34 (0.7)	27	1.54 (0.71)	0.371 *
FT3 [pg/mL]	18	2.84 (0.46)	27	3.24 (0.56)	0.014 *
FT4 [ng/dL]	18	1.22 (0.16)	27	1.28 (0.19)	0.239 *
TV [mL]	18	11.55 (9.1; 13.1)	26	13.45 (10.9; 19.1)	0.13 **

Abbreviations: ABI mean—mean ankle-brachial index value (taken from right side and left side values); TBI mean—mean toe-brachial index value (taken from right side and left side values); TSH—thyrotropin; FT3—free triiodothyronine; FT4—free thyroxine; SD—standard deviation; N—number of subjects; Q1—first quartile; Q3—third quartile; (*)—*p*-value according to the Student’s *t*-test; (**)—*p*-value according to the U Mann-Whitney test.

**Table 6:** The results of the Spearman’s rank correlation test between selected derivatives from thyroid volume and function parameters and vascular parameters.

Parameter	FT3/FT4		FT4 × TSH		FT4 × TSH × TV		FT3 × FT4 × TSH × TV	
	*R*	*p*	*R*	*p*	*R*	*p*	*R*	*p*
ABI right	0.169	0.268	−0.212	0.162	−0.069	0.655	−0.006	0.971
ABI left	0.073	0.633	−0.02	0.896	0.082	0.598	0.127	0.411
ABI mean	0.137	0.369	−0.116	0.447	0.017	0.911	0.07	0.653
ABI max	0.186	0.22	−0.058	0.704	0.051	0.744	0.114	0.46
ABI min	0.085	0.58	−0.179	0.239	−0.04	0.798	0.013	0.931
TP right	0.165	0.283	−0.034	0.826	0.228	0.142	0.302	0.049
TP left	0.129	0.404	0.126	0.415	0.361	0.017	0.378	0.013
TP mean	0.163	0.29	0.064	0.68	0.363	0.017	0.409	0.006
TP max	0.273	0.073	0.143	0.355	0.415	0.006	0.457	0.002
TP min	0.01	0.95	−0.057	0.715	0.212	0.173	0.264	0.087
TBI right	0.24	0.113	0.192	0.206	0.414	0.005	0.508	<0.001
TBI left	0.123	0.42	0.304	0.043	0.502	<0.001	0.544	<0.001
TBI mean	0.184	0.225	0.274	0.069	0.521	<0.001	0.592	<0.001
TBI max	0.271	0.072	0.322	0.031	0.544	<0.001	0.603	<0.001
TBI min	0.028	0.857	0.208	0.171	0.451	0.002	0.527	<0.001

Abbreviations: ABI right—ankle-brachial index measured on the right side; ABI left—ankle-brachial index measured on the left side; ABI mean—mean ankle-brachial index value (taken from right side and left side values); ABI max—maximal ankle-brachial index value (taken from right side and left side values); ABI min—minimal ankle-brachial index value (taken from right side and left side values); TP right—toe pressure measured on the right side; TP left—toe pressure measured on the left side; TP mean—mean toe pressure value (taken from right side and left side values); TP max—maximal toe pressure value (taken from right side and left side values); TP min—minimal toe pressure value (taken from right side and left side values); TBI right—toe-brachial index measured on the right side; TBI left—toe-brachial index measured on the left side; TBI mean—mean toe-brachial index value (taken from right side and left side values); TBI max—maximal toe-brachial index value (taken from right side and left side values); TBI min—minimal toe-brachial index value (taken from right side and left side values); TSH—thyrotropin; FT3—free triiodothyronine; FT4—free thyroxine; TV—thyroid volume. Statistically significant correlations are marked in red.

**Table 7:** Differences between patients with and without arterial hypertension.

Parameter	Patients with Arterial Hypertension		Patients without Arterial Hypertension		*p*
	N	Mean (SD)/Median (Q1; Q3)	N	Mean (SD)/Median (Q1; Q3)	
ABI right	19	1.13 (1.12; 1.27)	26	1.17 (1.08; 1.21)	0.765 **
ABI left	19	1.15 (1.07; 1.22)	26	1.15 (1.12; 1.2)	0.704 **
ABI mean	19	1.16 (1.1; 1.23)	26	1.16 (1.1; 1.21)	0.954 **
ABI max	19	1.18 (1.12; 1.27)	26	1.17 (1.12; 1.23)	0.73 **
ABI min	19	1.13 (1.07; 1.16)	26	1.15 (1.08; 1.2)	0.67 **
TP right [mmHg]	18	111.11 (30.67)	26	95.7 (20.12)	0.05 *
TP left [mmHg]	18	108.56 (26.42)	26	100.42 (23.39)	0.288 *
TP mean [mmHg]	18	109.83 (25.8)	26	98.06 (20.23)	0.097 *
TP max [mmHg]	18	119.17 (31.43)	26	104.7 (21.57)	0.077 *
TP min [mmHg]	18	100.5 (21.67)	26	91.42 (20.17)	0.16 *
TBI right	19	0.86 (0.25)	26	0.81 (0.17)	0.39 *
TBI left	19	0.83 (0.2)	26	0.85 (0.2)	0.789 *
TBI mean	19	0.85 (0.21)	26	0.83 (0.17)	0.74 *
TBI max	19	0.92 (0.24)	26	0.89 (0.19)	0.615 *
TBI min	19	0.77 (0.18)	26	0.77 (0.16)	0.928 *
TSH [µIU/mL]	19	1.28 (0.49)	26	1.59 (0.81)	0.143 *
FT3 [pg/mL]	19	3.23 (0.48)	26	2.98 (0.59)	0.137 *
FT4 [ng/dL]	19	1.26 (0.19)	26	1.25 (0.18)	0.941 *
TV [mL]	19	15.8 (11.4; 21.8)	25	11.7 (9.1; 13.1)	0.018 **

Abbreviations: ABI right—ankle-brachial index measured on the right side; ABI left—ankle-brachial index measured on the left side; ABI mean—mean ankle-brachial index value (taken from right side and left side values); ABI max—maximal ankle-brachial index value (taken from right side and left side values); ABI min—minimal ankle-brachial index value (taken from right side and left side values); TP right—toe pressure measured on the right side; TP left—toe pressure measured on the left side; TP mean—mean toe pressure value (taken from right side and left side values); TP max—maximal toe pressure value (taken from right side and left side values); TP min—minimal toe pressure value (taken from right side and left side values); TBI right—toe-brachial index measured on the right side; TBI left—toe-brachial index measured on the left side; TBI mean—mean toe-brachial index value (taken from right side and left side values); TBI max—maximal toe-brachial index value (taken from right side and left side values); TBI min—minimal toe-brachial index value (taken from right side and left side values); TSH—thyrotropin; FT3—free triiodothyronine; FT4—free thyroxine; TV—thyroid volume; (*)—*p*-value according to the Student’s *t*-test; (**)—*p*-value according to the U Mann-Whitney test.

**Table 8:** Differences between patients with diabetes mellitus or prediabetes and patients without abnormalities in carbohydrate metabolism.

Parameter	Patients with Diabetes or Prediabetes		Patients with No Abnormalities of Carbohydrate Metabolism		*p*
	N	Mean (SD)/Median (Q1; Q3)	N	Mean (SD)/Median (Q1; Q3)	
ABI right	9	1.13 (1.12; 1.18)	36	1.17 (1.08; 1.23)	0.287 **
ABI left	9	1.13 (1.1; 1.22)	36	1.15 (1.11; 1.2)	0.6 **
ABI mean	9	1.14 (1.11; 1.19)	36	1.17 (1.1; 1.22)	0.387 **
ABI max	9	1.15 (1.12; 1.22)	36	1.18 (1.12; 1.25)	0.342 **
ABI min	9	1.13 (1.1; 1.13)	36	1.15 (1.08; 1.2)	0.435 **
TP right [mmHg]	9	104.0 (93.0; 113.0)	35	105.0 (84.0; 118.0)	0.793 **
TP left [mmHg]	9	102.0 (85.0; 121.0)	35	105.0 (84.0; 122.0)	0.942 **
TP mean [mmHg]	9	107.5 (89.0; 116.5)	35	110.0 (80.5; 117.0)	0.919 **
TP max [mmHg]	9	113.0 (93.0; 128.0)	35	115.0 (92.0; 127.0)	1.0 **
TP min [mmHg]	9	102.0 (85.0; 105.0)	35	100.0 (76.0; 110.0)	0.8 **
TBI right	9	0.85 (0.69; 0.91)	36	0.84 (0.7; 0.99)	0.629 **
TBI left	9	0.78 (0.63; 1.03)	36	0.82 (0.71; 1.0)	0.691 **
TBI mean	9	0.87 (0.66; 0.96)	36	0.87 (0.68; 1.0)	0.65 **
TBI max	9	0.95 (0.69; 1.03)	36	0.89 (0.75; 1.06)	0.6 **
TBI min	9	0.78 (0.63; 0.89)	36	0.81 (0.65; 0.87)	0.64 **
TSH [µIU/mL]	9	1.37 (1.1; 1.58)	36	1.4 (0.997; 2.03)	0.798 **
FT3 [pg/mL]	9	3.01 (0.31)	36	3.1 (0.6)	0.671 *
FT4 [ng/dL]	9	1.19 (0.16)	36	1.27 (0.18)	0.216 *
TV [mL]	9	13.1 (11.7; 21.5)	35	12.5 (9.1; 16.1)	0.221 **

Abbreviations: ABI right—ankle-brachial index measured on the right side; ABI left—ankle-brachial index measured on the left side; ABI mean—mean ankle-brachial index value (taken from right side and left side values); ABI max—maximal ankle-brachial index value (taken from right side and left side values); ABI min—minimal ankle-brachial index value (taken from right side and left side values); TP right—toe pressure measured on the right side; TP left—toe pressure measured on the left side; TP mean—mean toe pressure value (taken from right side and left side values); TP max—maximal toe pressure value (taken from right side and left side values); TP min—minimal toe pressure value (taken from right side and left side values); TBI right—toe-brachial index measured on the right side; TBI left—toe-brachial index measured on the left side; TBI mean—mean toe-brachial index value (taken from right side and left side values); TBI max—maximal toe-brachial index value (taken from right side and left side values); TBI min—minimal toe-brachial index value (taken from right side and left side values); TSH—thyrotropin; FT3—free triiodothyronine; FT4—free thyroxine; TV—thyroid volume; (*)—*p*-value according to the Student’s *t*-test; (**)—*p*-value according to the U Mann-Whitney test.

**Table 9:** Differences between patients with BMI < 25.0 kg/m^2^ and patients with BMI ≥ 25.0 kg/m^2^.

Parameter	BMI < 25.0 kg/m^2^		BMI ≥ 25.0 kg/m^2^		*p*
	N	Mean (SD)/Median (Q1; Q3)	N	Mean (SD)/Median (Q1; Q3)	
ABI right	19	1.11 (1.05; 1.21)	26	1.18 (1.13; 1.23)	0.124 **
ABI left	19	1.14 (1.03; 1.19)	26	1.15 (1.1; 1.22)	0.476 **
ABI mean	19	1.12 (1.05; 1.18)	26	1.17 (1.13; 1.22)	0.237 **
ABI max	19	1.15 (1.07; 1.23)	26	1.19 (1.13; 1.25)	0.306 **
ABI min	19	1.11 (1.02; 1.17)	26	1.15 (1.1; 1.2)	0.194 **
TP right [mmHg]	19	88.9 (23.3)	25	111.96 (23.39)	0.002 *
TP left [mmHg]	19	94.5 (23.66)	25	110.76 (23.56)	0.029 *
TP mean [mmHg]	19	91.71 (21.82)	25	111.36 (20.7)	0.004 *
TP max [mmHg]	19	99.05 (23.95)	25	119.4 (25.71)	0.011 *
TP min [mmHg]	19	84.37 (20.79)	25	103.32 (17.53)	0.002 *
TBI right	19	0.76 (0.23)	26	0.88 (0.17)	0.073 *
TBI left	19	0.82 (0.23)	26	0.86 (0.18)	0.56 *
TBI mean	19	0.79 (0.22)	26	0.87 (0.15)	0.191 *
TBI max	19	0.86 (0.24)	26	0.93 (0.19)	0.277 *
TBI min	19	0.73 (0.2)	26	0.8 (0.13)	0.136 *

Abbreviations: ABI right—ankle-brachial index measured on the right side; ABI left—ankle-brachial index measured on the left side; ABI mean—mean ankle-brachial index value (taken from right side and left side values); ABI max—maximal ankle-brachial index value (taken from right side and left side values); ABI min—minimal ankle-brachial index value (taken from right side and left side values); TP right—toe pressure measured on the right side; TP left—toe pressure measured on the left side; TP mean—mean toe pressure value (taken from right side and left side values); TP max—maximal toe pressure value (taken from right side and left side values); TP min—minimal toe pressure value (taken from right side and left side values); TBI right—toe-brachial index measured on the right side; TBI left—toe-brachial index measured on the left side; TBI mean—mean toe-brachial index value (taken from right side and left side values); TBI max—maximal toe-brachial index value (taken from right side and left side values); TBI min—minimal toe-brachial index value (taken from right side and left side values); (*)—*p*-value according to the Student’s *t*-test; (**)—*p*-value according to the U Mann-Whitney test.

**Table 10:** Differences between patients with metabolic syndrome and patients without metabolic syndrome.

Parameter	Patients with Metabolic Syndrome		Patients without Metabolic Syndrome		*p*
	N	Median (Q1; Q3)	N	Median (Q1; Q3)	
ABI right	13	1.13 (1.12; 1.2)	32	1.16 (1.08; 1.23)	0.634
ABI left	13	1.15 (1.1; 1.22)	32	1.15 (1.11; 1.2)	0.831
ABI mean	13	1.16 (1.11; 1.21)	32	1.16 (1.1; 1.22)	0.726
ABI max	13	1.18 (1.12; 1.23)	32	1.17 (1.12; 1.23)	0.763
ABI min	13	1.13 (1.1; 1.16)	32	1.14 (1.08; 1.2)	0.651
TP right [mmHg]	13	112.0 (102.0; 119.0)	31	99.0 (82.0; 116.0)	0.269
TP left [mmHg]	13	105.0 (99.0; 140.0)	31	104.0 (79.0; 120.0)	0.361
TP mean [mmHg]	13	112.5 (100.5; 125.0)	31	104.0 (80.0; 116.5)	0.247
TP max [mmHg]	13	121.0 (102.0; 140.0)	31	108.0 (90.0; 123.0)	0.173
TP min [mmHg]	13	104.0 (99.0; 110.0)	31	98.0 (74.0; 110.0)	0.463
TBI right	13	0.89 (0.83; 0.96)	32	0.83 (0.66; 0.93)	0.299
TBI left	13	0.9 (0.75; 1.03)	32	0.8 (0.71; 0.98)	0.548
TBI mean	13	0.94 (0.79; 1.0)	32	0.85 (0.68; 0.99)	0.374
TBI max	13	1.02 (0.83; 1.14)	32	0.88 (0.74; 1.04)	0.347
TBI min	13	0.85 (0.75; 0.9)	32	0.8 (0.64; 0.87)	0.499

Abbreviations: ABI right—ankle-brachial index measured on the right side; ABI left—ankle-brachial index measured on the left side; ABI mean—mean ankle-brachial index value (taken from right side and left side values); ABI max—maximal ankle-brachial index value (taken from right side and left side values); ABI min—minimal ankle-brachial index value (taken from right side and left side values); TP right—toe pressure measured on the right side; TP left—toe pressure measured on the left side; TP mean—mean toe pressure value (taken from right side and left side values); TP max—maximal toe pressure value (taken from right side and left side values); TP min—minimal toe pressure value (taken from right side and left side values); TBI right—toe-brachial index measured on the right side; TBI left—toe-brachial index measured on the left side; TBI mean—mean toe-brachial index value (taken from right side and left side values); TBI max—maximal toe-brachial index value (taken from right side and left side values); TBI min—minimal toe-brachial index value (taken from right side and left side values); *p*-value according to the U Mann-Whitney test.

## Data Availability

The data presented in this study are available upon request from the corresponding author.
